# Construction and Characterization of Recombinant HSV-1 Expressing Early Growth Response-1

**DOI:** 10.1155/2014/629641

**Published:** 2014

**Authors:** Gautam Bedadala, Feng Chen, Robert Figliozzi, Matthew Balish, Victor Hsia

**Affiliations:** Department of Pharmaceutical Sciences, School of Pharmacy, University of Maryland Eastern Shore, 1 College Backbone Road, Princess Anne, MD 21853, USA

## Abstract

Early Growth response-1 (Egr-1) is a transcription factor that possesses a variety of biological functions. It has been shown to regulate HSV-1 gene expression and replication in different cellular environments through the recruitment of distinct cofactor complexes. Previous studies demonstrated that Egr-1 can be induced by HSV-1 infection in corneal cells but the level was lower compared to other cell types. The primary goal of this report is to generate a recombinant HSV-1 constitutively expressing Egr-1 and to investigate the regulation of viral replication in different cell types or in animals with Egr-1 overexpression. The approach utilized was to introduce Egr-1 into the BAC system containing complete HSV-1 (F) genome. To assist in the insertion of Egr-1, a gene cassette was constructed that contains the Egr-1 gene flanked byloxP sites. In this clone Egr-1 is expressed under control of CMV immediate-early promoter followed by another gene cassette expressing the enhanced green fluorescent protein (EGFP) under the control of the elongation factor 1α (EF-1 α) promoter. The constructed recombinant viruses were completed containing the Egr-1 gene within the viral genome and the expression was characterized by qRT-PCR and Western blot analyses. Our results showed that Egr-1 transcript and protein can be generated and accumulated upon infection of recombinant virus in Vero and rabbit corneal cells SIRC. This unique virus therefore is useful for studying the effects of Egr-1 during HSV-1 replication and gene regulation in epithelial cells and neurons.

## 1. Introduction

Herpes simplex virus type-1 (HSV-1) has a large genome that can accommodate up to 30 kb of foreign sequences after deletion of a number of nonessential-for-replication genes [1].This feature makes HSV-1-based vectors appropriate for a range of purposes in the development of gene therapy protocols. A number of strategies have been developed to manipulate the HSV-1 genome. The accessibility of a bacterial artificial chromosome (BAC) molecular clone of HSV-1 allowed efficient and rapid mutagenesis of large DNA sequences cloned into BACs, which were previously restricted to relatively small plasmids in *E. coli* [2]. In addition*,* Red recombination using PCR-amplified selectable markers as well as *en passant* mutagenesis utilizing markerless DNA strategy facilitates the insertions, deletions, and single point substitutions on a BAC-cloned DNA sequence [3]. In most circumstances, specific insertions that do not interfere with virus functions, while allowing stable expression of foreign genes, are required for subsequent studies.

In this study, the BAC containing HSV-1 infectious clone (pYEbac102) was used as a foundation for construction [4]. The BAC portion was not essential for any vial functions or recovery of infectious virion after transfection into eukaryotic cells but provided advantages since loxP recombination sites conveniently flank the BAC backbone. The BAC was placed at the intergenic region between the HSV-1 U_L_3 and U_L_4 during the original construction of HSV-1 pYEbac102. The backbone is cleavable from the virus genome via coinfection with recombinant adenovirus, AxCANCre, expressing the Cre recombinase [2]. The method includes the coinsertion of a selectable or visual marker such as the gene coding for enhanced green fluorescence protein (EGFP). This marker can be easily removed from the viral genome in subsequent step, if necessary. This protocol is widely applicable for any gene of interest within the pYEbac102 plasmid resulting in the rapid isolation of HSV-1 recombinant viruses expressing the gene of interest.

## 2. Materials and Methods

### 2.1. Cells and Viruses

293 cells (Cat#: CRL-1573, ATCC, Manassas, VA) were used to produce stock of recombinant adenovirus AxCANCre, expressing Cre recombinase protein. BAC plasmid DNA of parental HSV-1 infectious molecular clone (pYEbac102) was a gift of Dr. K. G. Kousoulas from Louisiana State University. The green monkey kidney cells (Vero) (Cat#: CCL-81, ATCC, Manassas, VA) were used for reconstitution of infectious virus. Vero and 293 cells are grown in DMEM supplemented with 10% FBS according to the manufacturer. Nonrecombinant viruses used in the study were designated as A1: 17Syn^+^/EGFP [5] and A4: RE strain [6]. The recombinant virus generated in this study was named A7, which contains a cassette expressing Egr-1 under CMV promoter. Another recombinant virus A8 was generated independently overexpressing TRβ1 using same strategy but different cassette and this virus was used as a comparison control since it has exactly the same genetic background as A7.

### 2.2. Bacteria and Plasmids

All plasmids were generated in One Shot TOP10 *E. coli* grown at 37°C in Luria-Bertani (LB) medium (Invitrogen, Carlsbad, CA). Plasmid containing cloned EGFP gene sequence with the Lox P sites (EGFP Vector) was used as backbone for recombinant DNA construction and was kindly provided by Dr. K. G. Kousoulas (LSU, Baton Rouge). PCDNA3.1/V5-His-TOPO cloning vector (Invitrogen, Carlsbad, CA) was used for step-by-step construction of recombination cassette.

### 2.3. Primers and PCR

Egr-1 (1630 bp) sequence was amplified from Egr-1 over-expression vector using the primers: FORWARD: 5′-AGG ATG GCC GCG GCC AAG G-3′; REVERSE: 5′-GCA AAT TTC AAT TGT CCT GGG-3′ [7]. The forward primer was designed to recognize the upstream region of the starting codon. The reverse primer was designed in front of the stop codon to facilitate the expression of V5 epitope, when cloned into PC DNA 3.1/V5/HIS-TOPO. The PCR was carried out using Failsafe PCR enzyme Mix (Epicentre: Cat#: FS99250) and Buffer Mix E (FSP995E). The PCR condition was determined empirically with the annealing temperature at 58°C and elongation time of 1min 30 sec. The resulting product was named Egr-1 PCDNA 3.1. A second set of primers was designed such that the forward primer is just before the CMV promoter in the PCDNA 3.1 and has Cla1 restriction enzyme site. The reverse primer was designed to be located downstream to the polyadenylation signal and has Pac1 restriction enzyme site. The sequences for the primers were named CMV FOR: 5′-GCC GTC ATC GAT GAA GAA TCT GCT TAG GGT TAG-3′; and Poly A REV: 5′-CGC CAC CCG AGA TAC CGA AGA AAT TAA TTG AAT GG-3′. The restriction sites of Cla I and Pac I were marked underlined, respectively. PCR was performed using the previously cloned Egr-1 PCDNA 3.1 as template and by Failsafe enzyme Mix and Buffer Mix E. The PCR conditions were the same as described previously with minor modification of elongation time of 2min 30 sec. The size of the PCR product is 2.7 kb.

### 2.4. Cloning of Egr-1 into the Egfp Vector

The EGFP vector and the 2.7 kb PCR products were double-digested with enzymes Cla1 and Pac1 in the presence of 1X NEB buffer 4 and 1X BSA at 37°C overnight. The digestion products were gel-purified using Zymoclean gel extraction kit (D4001, Zymo Research, Irvine, CA). Ligation was performed using the vector/insert ratio of 1 : 5 in the presence of 1U T4 DNA ligase in 20 μL final volume overnight at room temperature. Transformation was done using 2 μL of the ligation mix with one-shot TOP10 competent cells described essentially by the manufacturer. Colonies were picked up for small culture followed by miniprep extraction. The validation was examined by double digestion of Cla1 and Pac1 and resolved by gel electrophoresis. The resulting plasmid is named pEgr1-EGFP.

### 2.5. Cre-Lox Recombination

**Figure 1:**
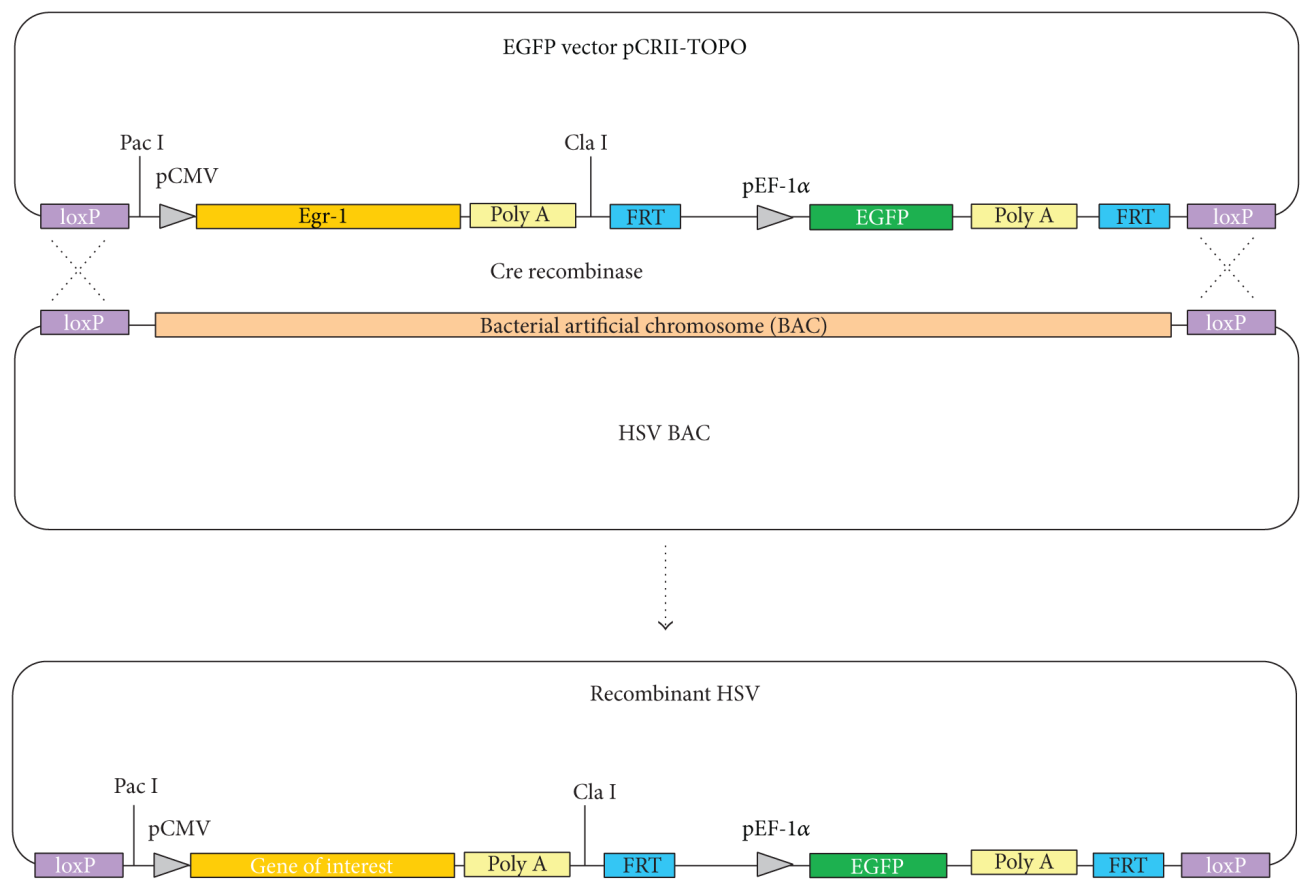
Schematic representation of the recombinant virus construction.

The scheme of recombination and construction of the chimeric virus was described (Figure 1). In short, 293 cells were transfected with 2 μg of pEgr1-EGFP using Lipofectamine transfection reagent (Invitrogen). At the 6 hrs after transfection the medium containing transfection complexes was removed/washed and the cells were immediately coinfected with HSV-1 BAC and adenovirus AxCANCre [8]. Different adenovirus concentrations ranging from 1 : 20 to 1 : 200 dilutions were tested. A total of 1mLof infectionmix solution (HSV-1/BACcombined with adenovirus) in DMEM media containing 25mM of HEPES was incubated with transfected cells for 1 h at room temperature. At different time points after infection (16 hpi, 22 hpi, and 36 hpi), aliquots of the supernatant media (containing HSV-1/BAC and recombinant HSV-1) were collected and utilized to infect fresh Vero cells.

### 2.6. Plaque Purification

**Figure 2:**
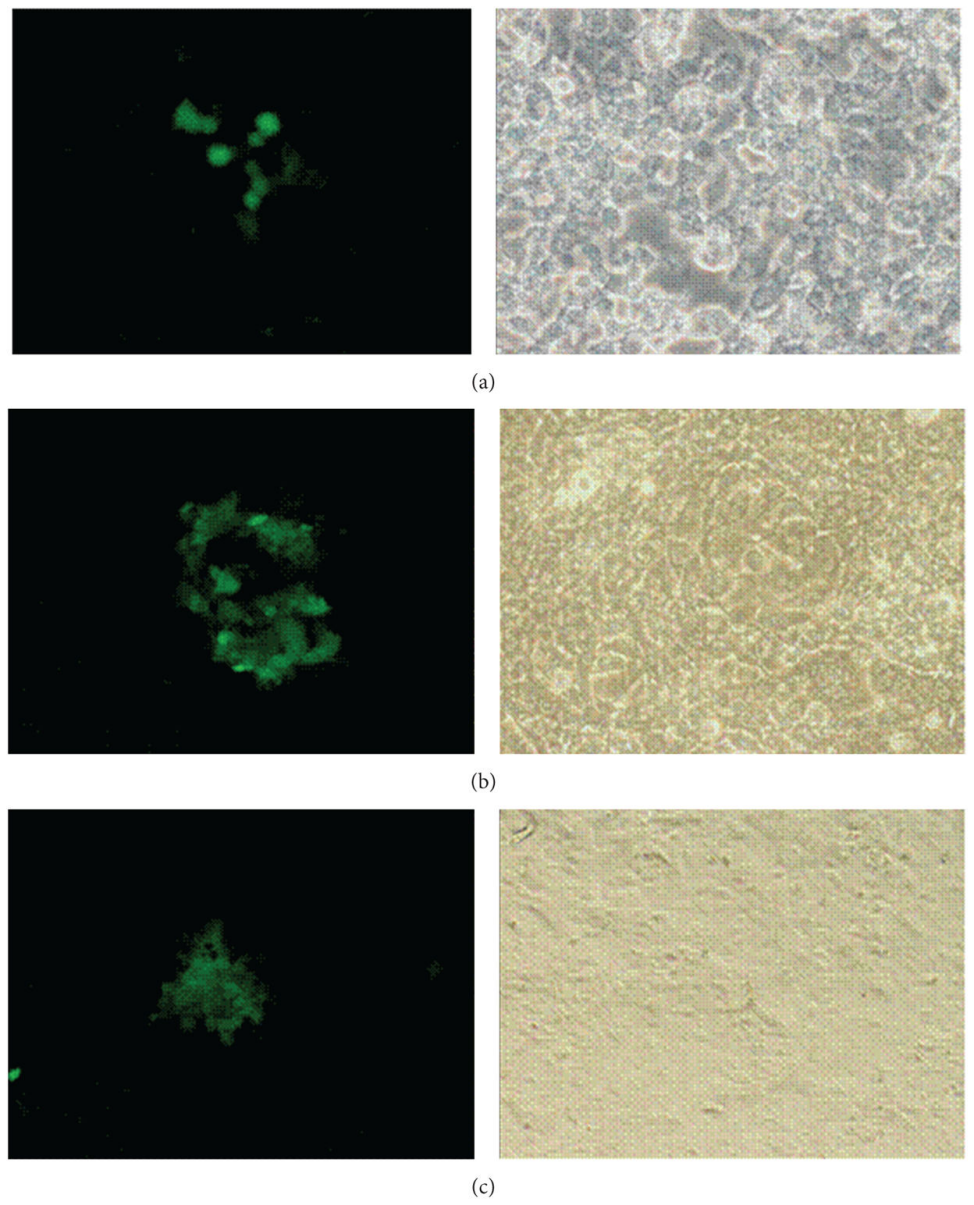
Plaque purification of the recombinant HSV-1. (a) First round of infection shows the presence of green plaque of recombinant HSV-1 surrounded by the plaques of regular HSV-1 BAC virus. (b) Second round of plaque purification was performed by collecting the plaque from round one followed by infecting fresh Vero cells in a dilution of 1 : 20. Decrease in the background by regular HSV-1 BAC virus can be seen. (c) Third round of plaque purification was done by repeating the round 2 and showed no background by the regular HSV-1 BAC virus.

Infection was performed for 1hr using medium without serum for making virus dilutions to obtain a moi of 2. At 22 hpi, the cells were placed in −80°C freezer for a day. Later, the medium was thawed, centrifuged, and used to infect fresh Vero cells at different dilutions. Infection was performed for 1 hr followed by removal of the virus and addition of medium containing methylcellulose. The composition of the media was as follows: 1% methylcellulose in DMEM, 15 nM sodium bicarbonate, 25 nM HEPES, and 2% FBS. At 24 hpi, cells were checked for green fluorescent signal. Any plaque with green fluorescence found was marked and collected. These plaques were used to infect fresh Vero cells in different dilutions for plaque purification. Three rounds of plaque purification were performed until the background from the regular virus disappeared (Figure 2).

### 2.7. Quantitative Analyses of the Egr-1 Overexpression

The overexpression of Egr-1 was evaluated by infection followed by quantitative RT-PCR (qRT-PCR). In summary, cells were infected by parental and recombinant virus at moi of 5. At different hpi, total RNA was isolated and purified by Trizol using manufacturer’s protocol (Life Technologies). For qRT-PCR, 0.5 μg of total RNA and primer pairs were assembled and subjected to iScript One-Step RT-PCR SYBR Green kit (Bio-Rad, Cat# 170-8892) operated on Bio-Rad MyiQ single-color real-time PCR detection system (Cat#: 170-9740). The fold of enrichment was assessed by measuring ΔΔCt normalized by GFP signal and cellular genes as internal and cellular controls, respectively. The sequences of the primers were as follows: Egr-1: 5′-AGA CCA GTT ACC CCA GCC AAA C-3′and 5′-AAA ATG TCA GTG TTC GGC GTG-3′; GFP: 5′-GCA GAA GAA CGG CAT CAA GGT G-3′and 5′-TGG GTG CTC AGG TAG TGG TTG TC-3′. The cellular control primers were as follows: PPIA 5′-AGC ATA CGG GTC CTG GCA TCT-3′and 5′-CAT GCT TGC CAT CCA ACC ACT CA-3′[9]; PGK1: 5′-ACC TGC TGG CTG GAT GGG CTT-3′and 5′-GCT TAG CCC GAG TGA CAG CCT C-3′[9]. The reaction was performed at 50°C for 20min and 94°C for 2min, and followed by 35 cycles of 94°C for 30s, 61°C for 30s, and 68°C for 30s.

### 2.8. Western Blotting

Our established protocol was described in [10]. In summary, cell extract was separated by gel electrophoresis and transferred to membrane using iBlot Gel Transfer Device (Cat#: IB1001) from Invitrogen (Carlsbad, CA). Anti-Egr-1 rabbit polyclonal antibody (Santa Cruz SC-110x) was added at a dilution of 1 : 1,000 for detection. Anti-α-Tubulin mouse antibody (Calbiochem, Cat#: CP06, San Diego, CA) was used at a dilution of 1 : 10,000 as control. The chemiluminescent signal from the membranes was detected by Bio-Rad Chemi-docl XRS imaging systems (Hercules, CA).

## Results

### 3.1. Recombination of BAC and pEgr-1-EGFP by AxCANCre

The key step of this protocol is the efficiency of recombination. Our results showed that 22 hpi supernatant produced up to 5 green fluorescent viral plaques per each well of a 6-well plate (data not shown). It is likely during the earlier time (16 hpi) that there is not enough of Cre recombinase protein can be expressed regardless of the concentration of recombinant adenovirus used. In contrast, during the later course of infection due to an excess of Cre enzyme, the recombination reaction was shifted toward excision of loxP flanked inserts and no recombinant green viruses were detected at 36 hpi. Green plaques were selected and subjected to 3 rounds of plaque isolation to ensure the purity (Figure 2).

### 3.2. Overexpression of Egr-1 by Recombinant Virus in SIRC Cells

**Figure 3:**
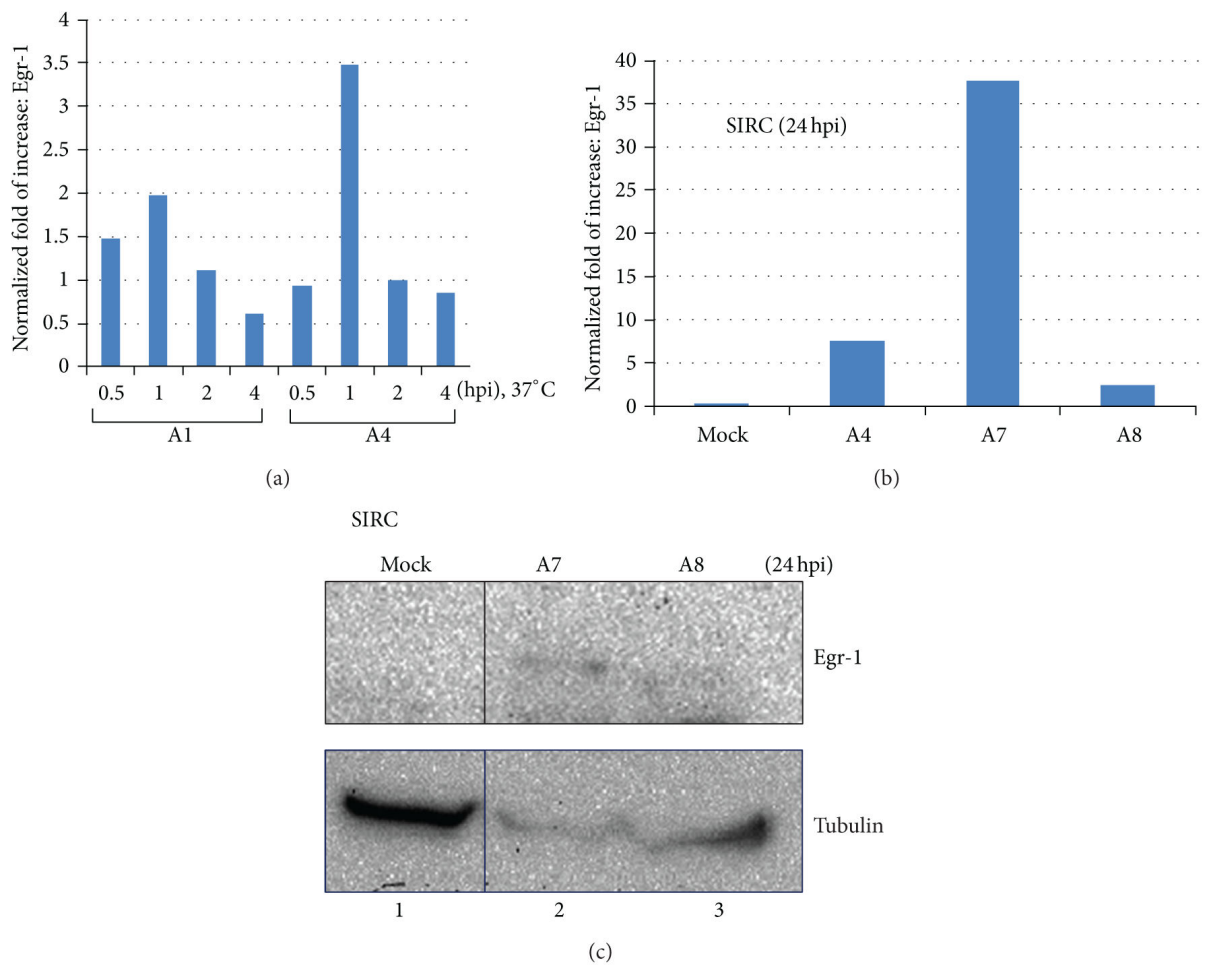
Expression profile of Egr-1 induced by infection of SIRC cells. (a) Disparity of Egr-1 induction by different virus strains. Cells were infected by viruses with moi of 5 at 4°C for 1 h for attachment followed by incubation at 37°C with various time points. Total RNA was isolated and subjected to qRT-PCR to measure the Egr-1 expression. (b) Accumulation of Egr-1 transcript from recombinant viruses. Total RNA from 24 h postinfected SIRC cells were purified followed by RT-PCR assays. (c) Overexpression of Egr-1 from recombinant virus. Western blot analyses were performed at the condition described in (b) using anti-Egr-1 Ab.

Egr-1 can be induced by HSV-1 infection and the protein was sufficient to be detected at 24 hpi in SIRC cells [10]. Interestingly, we showed that the Egr-1 transcript can be detected rapidly and peaked at 1hpi byA1 andA4 at 37°C and significantly decreased thereafter (Figure 3(a)). To address the capability of Egr-1 overexpression from recombinant virus A7, SIRC cells were infected by A4, A7, and A8 followed by RNA purification at 24 hpi at 37°C and the quantitative analyses of Egr-1 expression were assessed by qRT-PCR using primers against Egr-1 ORF and normalized by internal and cellular control genes PPIA1 and PGK1 [9]. The results showed that the cells while infected with A4 exhibited a 26-fold increase of Egr-1 expression whereas the recombinant virus A7 generated a 130-fold increase compared to no infection control (Figure 3(b)). It is noted that more than 15-fold of expression difference was observed between A7 and A8 infection, indicating the increase derived from the cassette expression (Figure 3(b)). To further confirm the expression at the translation level, Western blot analyses were performed using Egr-1 antibody and the results demonstrated the presence of full-length protein from A7 infected cells (Figure 3(c)). Together these findings demonstrated that recombinant virus A7 was successfully overexpressing Egr-1 in SIRC cells.

### 3.3. Induction of Egr-1 by Infection in Vero Cells

**Figure 4:**
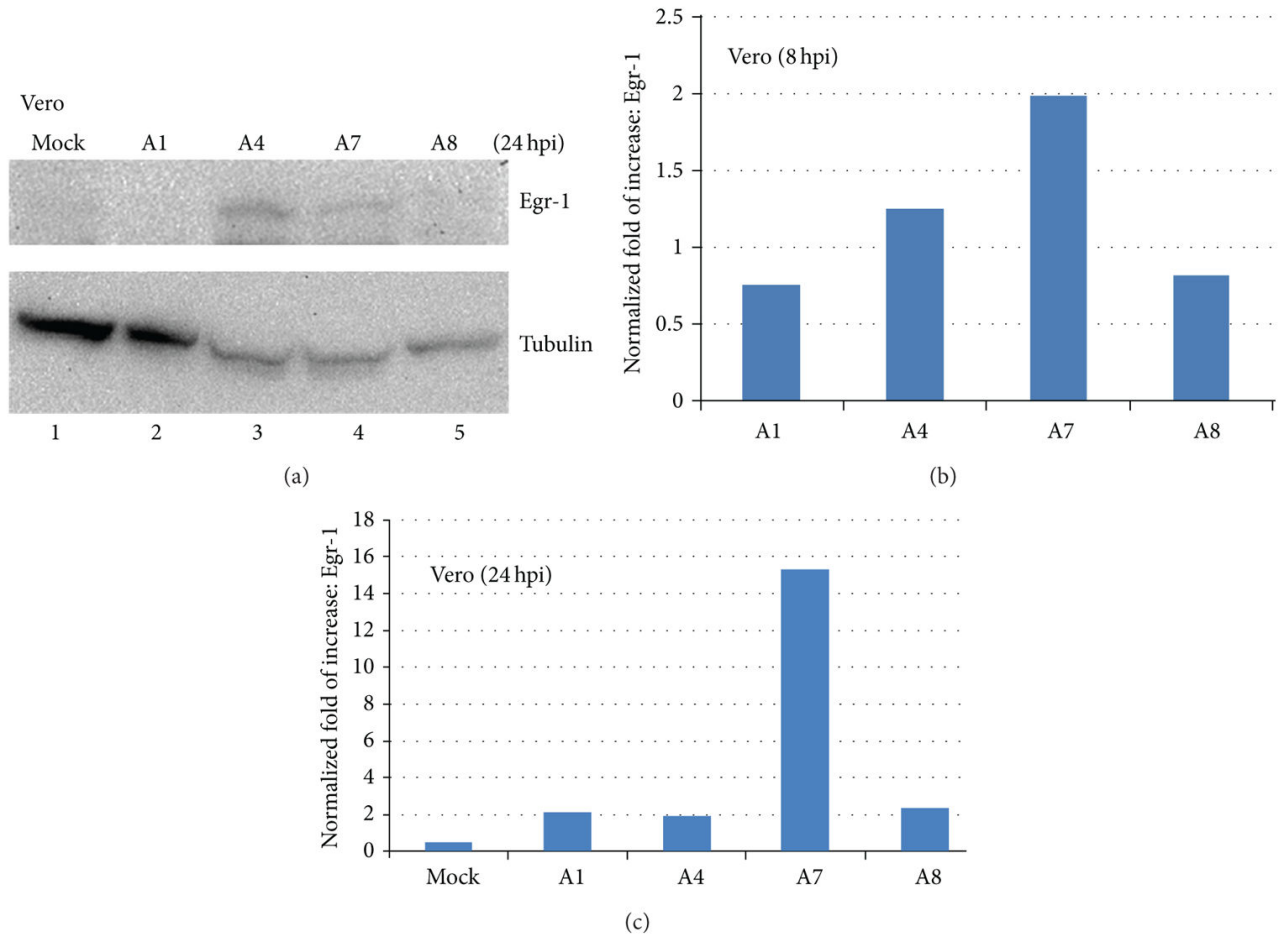
Expression profile of Egr-1 induced by infection of Vero cells. (a) Comparison of Egr-1 induction by different virus strains 24 h after infection of Vero cells. The condition of infection was the same described in Figure 3 with different cells. (b) Quantitative measurements of Egr-1 mRNA production by different viruses at 8 hpi. (c) Direct assessments of Egr-1 transcript accumulation at 24 hpi.

HSV mediated Egr-1 induction is complex in Vero cells. Our previous observation and current results revealed that only A4 can induce Egr-1 expression in this cell line (Figure 4(a), lane 3). A1 virus (17Syn^+^), however, failed to trigger the Egr-1 expression (Figure 4(a), lane 2). Recombinant virus A7, nonetheless, was sufficient to produce Egr-1 protein in Vero cells although weaker compared to A4 infection (Figure 4(a), lane 4). A8, another recombinant virus with same genetic background containing different cassette, produces no Egr-1 protein (Figure 4(a), lane 5). These results confirmed that Egr-1 can be overexpressed by recombinant virus in Vero cells and it was produced from the inserted cassette not from induction of infection. Additional experiments using qRT-PCR to measure the level of transcript showed distinct pattern of expression profile among those viruses. Our quantitative analyses indicated that A1, A4, and A8 viruses, in fact, generated similar level of Egr-1 mRNA at 8 and 24 hpi (Figure 4(b)). The recombinant virus A7, nevertheless, accumulated Egr-1 transcript approximately 8-foldmore than other viruses did at 24 hpi (Figure 4(c)). These results showed that although recombinant virus A7 continued to produce more Egr-1 transcript, the A4 virus exhibited much better efficiency to translate Egr-1 mRNA than A7 in Vero cells.

## 4. Conclusion

The Egr-1 induction by HSV-1 infection exhibited a multifaceted pattern. Previous observation demonstrated that Egr-1 transcript can be detected as early as one hour after the virus attachment to the cell membrane at 4°C without entry [10]. Our additional studies indicated that the accumulation of Egr-1 transcript reached its highest point at 1 hpi incubated at 37°C and diminished gradually (Figure 3(a)). The recombinant virus A7, on the other hand, continued to produce stable Egr-1mRNA detected at 24 hpi (Figures 3(b) and 4(c)). Further studies from Figures 3(c) and 4(a) indicated that accumulation of Egr-1 mRNA was translated into proteins. These observations suggested that the nascent Egr-1 overexpression from recombinant virus generates a steady increase of Egr-1 mRNA. The endogenous Egr-1 mRNA, nevertheless, possesses a unique regulatory mechanism at transcriptional and post-transcriptional levels to ensure a rapid turnover rate within infected cells in response to viral assault.

The half-life of endogenous Egr-1 transcript induced by A4 infection is short since very low level of Egr-1 mRNA can be detected at 24hpi (Figure 4(c)). Interestingly, the amount of protein was abundant at 24 hpi. The recombinant virus A7, although it generated approximately 8-fold increase of Egr-1 mRNA at 24 hpi compared to A4 strain, did not produce equivalent level of protein (Figure 4(a)). It is not clear why there is a discrepancy in Egr-1 transcription and translation, but it is likely due to high translation efficiency from the endogenous transcript. Additional experiments are underway to investigate this novel observation.

This rapid induction prompted us to hypothesize that the binding of the virus to target cells may trigger the protein synthesis. However, our results did not support the hypothesis since the infection at 4°C in which viruses attached without entry showed no evidence of Egr-1 induction (data not shown), suggesting that episodes after entry are required for successful protein expression. Additional observations using UV-inactivated viruses indicated that *de novo* viral protein synthesis is necessary for this unique induction. To investigate the contribution of viral replication in this induction, ACV was used to block the HSV-1 DNA synthesis and late viral gene expression. The results revealed clear sign of Egr-1 expression [11]. Together, these observations strongly suggest that Egr-1 induction requires HSV-1 protein expression prior to the viral DNA replication, and tegument proteins, if involved, are not sufficient. Nevertheless, our preliminary data revealed that partial Egr-1 transcript was detected upon viral infection at 4°C or by UV-inactivated viruses (data not shown). The mechanisms are not understood but it is likely that viral proteins facilitated the Egr-1 translation or prevented premature transcription termination. More studies are underway to investigate this novel phenomenon.

## Abbreviation

Egr-1: Early growth response protein 1
HSV-1: Herpes simplex virus type 1
BAC: Bacterial artificial chromosome
IE: Immediate-early gene
moi: Multiplicity of infection
hpi: Hours post infection
U_L_3: Unique Long 3
U_L_4: Unique Long 4

## References

[1] Guffey MB, Parker JN, Luckett WS (2007). Engineered herpes simplex virus expressing bacterial cytosine deaminase for experimental therapy of brain tumors. Cancer Gene Therapy.

[2] Tanaka M, Kagawa H, Yamanashi Y, Sata T, Kawaguchi Y (2003). Construction of an excisable bacterial artificial chromosome containing a full-length infectious clone of herpes simplex virus type 1: viruses reconstituted from the clone exhibit wild-type properties in vitro and in vivo. Journal of Virology.

[3] Murphy KC (1998). Use of bacteriophage λ recombination functions to promote gene replacement in *Escherichia coli*. Journal of Bacteriology.

[4] Melancon JM, Luna RE, Foster TP, Kousoulas KG (2005). Herpes simplex virus type 1 gK is required for gB-mediated virus-induced cell fusion, while neither gB and gK nor gB and UL20p function redundantly in virion de-envelopment. Journal of Virology.

[5] Foster TP, Rybachuk GV, Kousoulas KG (1998). Expression of the enhanced green fluorescent protein by herpes simplex virus type 1 (HSV-1) as an in vitro or in vivo marker for virus entry and replication. Journal of Virological Methods.

[6] Kaufman HE, Varnell ED, Thompson HW (1999). Cidofovir and experimental herpetic stromal disease. Archives of Ophthalmology.

[7] Bedadala GR, Pinnoji RC, Hsia S-CV (2007). Early Growth Response gene 1 (Egr-1) regulates HSV-1 ICP4 and ICP22 gene expression. Cell Research.

[8] Sato N, Wang S, Li L (1998). A novel strategy for introducing exogenous Bcl-2 into neuronal cells: the Cre/loxP system-mediated activation of Bcl-2 for preventing programmed cell death using recombinant adenoviruses. Molecular and Cellular Neurosciences.

[9] Watson S, Mercier S, Bye C, Wilkinson J, Cunningham AL, Harman AN (2007). Determination of suitable housekeeping genes for normalisation of quantitative real time PCR analysis of cells infected with human immunodeficiency virus and herpes viruses. Virology Journal.

[10] Bedadala GR, Palem JR, Graham L, Hill JM, McFerrin HE, Hsia S-C (2011). Lytic HSV-1 infection induces the multifunctional transcription factor Early Growth Response-1 (EGR-1) in rabbit corneal cells. Virology Journal.

[11] Hsia SC, Graham LG, Bedadala GR, Balish MB, Chen F, Figliozzi RW (2013). Induction of transcription factor early growth response protein 1 during HSV-1 infection promotes viral replication in corneal cells. British Microbiology Research Journal.

